# Lorlatinib als vielversprechendes Medikament in der zukünftigen Therapie des fortgeschrittenen ALK-positiven nichtkleinzelligen Bronchialkarzinoms

**DOI:** 10.1007/s00066-021-01788-z

**Published:** 2021-06-10

**Authors:** Johanna Richter, Jürgen Dunst

**Affiliations:** 1 Strahlentherapie und Onkologie, Christian-Albrechts-Universität zu Kiel, Kiel, Deutschland; 2 Klinik für Strahlentherapie, Campus Kiel, Universitätsklinikum Schleswig-Holstein, Feldstr. 21, 24105 Kiel, Deutschland

## Hintergrund

Zielgerichtete Therapien haben in den letzten Jahren die Therapie des nichtkleinzelligen Bronchialkarzinoms (NSCLC) drastisch verändert. Ein besseres Verständnis der Pathogenese ermöglichte die Entwicklung von Tyrosinkinaseinhibitoren, die spezifisch in die Signalwege von Treibermutationen eingreifen [1]. Eine dieser Treibermutationen beim NSCLC ist das ALK-Rearrangement (Anaplastic Lymphoma Kinase). Ein solches Rearrangement liegt bei ca. 7 % aller Patienten mit NSCLC vor [2]. Mit der Entwicklung von Crizotinib wurde der erste zielgerichtete Tyrosinkinaseinhibitor für Patienten mit einem ALK-positiven Bronchialkarzinom hergestellt [1]. Trotz des Erfolgs wurde schon nach kurzer Zeit das Auftreten von Resistenzen unter Crizotinibtherapie beschrieben [3]. Es folgte die Entwicklung mehrerer Tyrosinkinaseinhibitoren der zweiten Generation wie Alectinib und Brigatinib. Auch unter der Therapie mit den weiterentwickelten Tyrosinkinaseinhibitoren kam es zu Resistenzbildung. Lorlatinib, ein Tyrosinkinaseinhibitor der dritten Generation, zeigte eine breitere Wirksamkeit auch beim Vorliegen von Resistenzmutation gegen Tyrosinkinaseinhibitoren der zweiten Generation sowie eine besonders hohe ZNS-Wirksamkeit [4]. Die CROWN-Studie als globale, prospektive, randomisierte Phase-III-Studie untersuchte nun Lorlatinib im Vergleich mit Crizotinib als Therapie von Patienten mit unbehandelten, fortgeschrittenen ALK-positiven nichtkleinzelligen Bronchialkarzinomen.

## Patientengut und Methoden

In die Studie eingeschlossen wurden Patienten mit fortgeschrittenen bzw. metastasierten ALK-positiven NSCLC (Stadium IIIB bzw. IV), die zuvor nicht systemisch behandelt worden waren. Die rekrutierten Patienten mussten mindestens eine extrakranielle vorher nicht bestrahlte Manifestation aufweisen. Eingeschlossene Patienten zeigten einen ECOG-Status zwischen 0 und 2 sowie eine dem Protokoll entsprechend ausreichende Knochenmarks‑, Pankreas‑, Nieren- als auch Leberfunktion. Je nach Gruppenzuordnung erhielten die Patienten entweder 100 mg Lorlatinib einmal täglich oder 250 mg Crizotinib zweimal täglich. Ab dem Zeitpunkt der Randomisierung erfolgte eine Beurteilung des Tumors alle 8 Wochen anhand von CT- bzw. MRT-Aufnahmen des Thorax, Abdomens und Beckens. Zusätzlich erfolgte unabhängig vom ZNS-Befall stets eine MRT-Bildgebung des Kopfes. Als primärer Endpunkt der Studie wurde das progressionsfreie Überleben (PFS) festgelegt. Sekundäre Endpunkte waren Gesamtüberleben (OS), objektives Ansprechen bei ZNS-Befall sowie die Verträglichkeit des Medikaments.

## Ergebnisse

Von März 2017 bis Februar 2019 konnten insgesamt 296 Patienten rekrutiert werden. Im Rahmen der Randomisierung wurden 149 Patienten der Lorlatinibgruppe und 147 Patienten der Crizotinibgruppe zugeordnet. Zu Beginn der Studie lag bei 26 % der Patienten in der Lorlatinibgruppe sowie bei 27 % der Patienten der Crizotinibgruppe eine Gehirnmetastasierung vor. Im Rahmen einer geplanten Zwischenanalyse wurden die Daten nach Eintritt von 75 % aller zu erwartenden Ereignisse ausgewertet. Zum Zeitpunkt der Zwischenanalyse war es bei insgesamt 127 Patienten entweder zu einem Krankheitsfortschritt oder zum Tod gekommen. Innerhalb der Lorlatinibgruppe kam es bei 41 der 149 Patienten (28 %) zu einem derartigen Ereignis, während es innerhalb der Crizotinibgruppe 86 der 147 Patienten (59 %) betraf.

Nach 12 Monaten waren 78 % (95 %-KI 70–84 %) der Patienten der Lorlatinibgruppe noch am Leben ohne weiteren Krankheitsfortschritt, während es in der Crizotinibgruppe nur 39 % (95 %-KI 30–48 %) waren. Das mittlere progressionsfreie Überleben betrug 18,3 Monate in der Lorlatinibgruppe und 14,8 Monate in der Crizotinibgruppe. Die Hazard Ratio im Hinblick auf Krankheitsfortschritt oder Tod lag mit 0,28 (95 %-KI 0,19–0,41; *p* < 0,001) zugunsten der Loratinibgruppe. Ein objektives Ansprechen zeigte sich bei 70 % in der Lorlatinibgruppe und nur bei 27 % innerhalb der Crizotinibgruppe. Zum Zeitpunkt der Datenanalyse konnte noch kein signifikanter Unterschied zwischen den beiden Gruppen im Gesamtüberleben festgestellt werden.

Unter den Patienten mit messbarer Gehirnmetastasierung zum Ausgangszeitpunkt zeigten innerhalb der Lorlatinibgruppe 82 % ein intrakranielles Ansprechen und 72 % eine intrakranielle Komplettremission. Ein anhaltendes intrakranielles Ansprechen für mindestens 12 Monate konnte bei 72 % der Patienten der Lorlatinibgruppe erreicht werden, während dies bei keinem Patienten der Crizotinibgruppe der Fall war. Nur 3 % der Patienten in der Lorlatinibgruppe zeigten unter der Therapie ein Fortschreiten der Gehirnmetastasierung, hingegen 33 % der Crizotinibpatienten.

Unter Lorlatinibeinnahme traten folgende unerwünschte therapiebedingte Ereignisse häufiger als unter Einnahme von Crizotinib auf: Hypercholesterinämie, Hypertriglyzeridämie, Ödeme, Gewichtszunahme, periphere Neuropathie, kognitive Nebenwirkungen, Anämie, Bluthochdruck, Stimmungsveränderungen sowie Hyperlipidämie. In der Lorlatinibgruppe wurden häufiger höhergradige Nebenwirkungen (CTCAE-Grad 3 oder 4) erfasst als in der Crizotinibgruppe. Unter der Therapie kam es im Rahmen unerwünschter Ereignisse in beiden Gruppen bei je 5 Patienten zum Tod. In der Lorlatinibgruppe wurden 2 dieser Todesfälle als möglicherweise therapieassoziiert eingestuft. Unerwünschte therapieassoziierte Ereignisse, die zu einem Therapieabbruch führten, lagen bei 7 % der Patienten der Lorlatinibgruppe und 9 % der Patienten der Crizotinibgruppe vor.

## Schlussfolgerung der Autoren

Patienten der Lorlatinibgruppe erleben insgesamt eine wesentliche Verbesserung der allgemeinen Lebensqualität im Vergleich zur Ausgangslage und den Patienten der Crizotinibgruppe.

## Kommentar

Bisher wurde Lorlatinib in der Zweitlinie nach erfolgloser Therapie mit Tyrosinkinaseinhibitoren der ersten bzw. zweiten Generation zugelassen. Wie präklinische sowie Phase-I- und -II-Studien vermuten ließen, zeigte sich Lorlatinib auch in den Zwischenergebnissen der Phase-III-Studie überlegen gegenüber der Therapie mit Crizotinib, dem Tyrosinkinaseinhibitor der ersten Generation. Die inzwischen entwickelten Tyrosinkinaseinhibitoren der zweiten Generation konnten in Studien jedoch bereits ebenfalls eine Überlegenheit gegenüber Crizotinib zeigen [5, 6]. Obwohl ein direkter Vergleich zwischen verschiedenen Studien von Natur aus aufgrund des unterschiedlichen Studienaufbaus sowie untersuchter Populationen nur eingeschränkte Aussagekraft besitzt, zeigte Lorlatinib einen mindestens genauso großen Effekt im Vergleich zu Crizotinib wie Tyrosinkinaseinhibitoren der zweiten Generation. Das PFS nach 12 Monaten lag mit 78 % bei Lorlatinib sogar oberhalb des PFS unter Alectinib oder Brigatinib mit 68,4 % bzw. 67 %. Verglichen mit der Crizotinibtherapie zeigte Lorlatinib mit einer Hazard Ratio von 0,28 ein 72 %ig niedrigeres Risiko für eine Krankheitsprogression oder Tod. Alectinib und Brigatinib präsentierten im Vergleich zur Crizotinibtherapie nur ein 53 %ig bzw. 51 %ig niedrigeres Risiko für ein solches Ereignis [5, 6].

Eine Metastasierung ins Gehirn ist ein häufiges und prognosebestimmendes Problem innerhalb der Gruppe des ALK-positiven NSCLC [7]. Lorlatinib zeigte im Vergleich zu Crizotinib eine überlegene Wirksamkeit auf eine vorliegende Gehirnmetastasierung. Vorangegangene Studien zu Crizotinib hatten bereits eine schlechte Überwindung der Blut-Hirn-Schranke festgestellt [8]. Lorlatinib wurde als Tyrosinkinaseinhibitor der dritten Generation gezielt mit Eigenschaften für eine hohe intrakranielle Verfügbarkeit entwickelt. Auch Tyrosinkinaseinhibitoren der zweiten Generation weisen ein besseres intrakranielles Ansprechen im Vergleich zu Crizotinib auf. Die Ansprechraten messbarer Gehirnmetastasen auf die Therapie mit Alectinib bzw. Brigatinib zeigten sich vergleichbar mit dem in der CROWN-Studie erreichten Ansprechen unter Lorlatinib. Trotz vergleichbarer Ansprechraten präsentierte Lorlatinib höhere Raten an intrakraniellen Komplettremissionen im Vergleich zu Alectinib und Brigatinib [5, 6]. Es werden weitere Studien benötigt, um die Wirksamkeit von Lorlatinib im Vergleich zu Tyrosinkinaseinhibitoren der zweiten Generation zu erkennen.

Die hohen intrakraniellen Ansprechraten der weiterentwickelten Tyrosinkinaseinhibitoren werfen zwangsläufig die Frage auf, welche Rolle die Strahlentherapie in der künftigen Behandlung des fortgeschrittenen NSCLC mit ZNS-Befall spielt. Durch den Einsatz weiterentwickelter Tyrosinkinaseinhibitoren wie Lorlatinib in der Erstlinie könnte möglicherweise eine Ganzhirnbestrahlung erst zeitlich verzögert oder überhaupt ganz vermieden werden [8]. Auch könnten durch Tyrosinkinaseinhibitoren Therapiesituationen erreicht werden, die wieder für die besser verträgliche stereotaktische Radiochirurgie zugänglich sind [7].

Im Vergleich zu Crizotinib zeigte Lorlatinib häufiger höhergradige unerwünschte Nebenwirkungen. Das Nebenwirkungsprofil deckte sich mit den bereits unter Lorlatinib beschriebenen Ereignissen. Bei der doch recht hohen Rate an schwerwiegenden Nebenwirkungen gilt es jedoch zu beachten, dass es sich bei über der Hälfte dieser Fälle um erhöhte Werte für Cholesterin, Triglyzeride oder beides handelte, beide können erfolgreich medikamentös behandelt werden. Kritischer hingegen sind die kognitiven Nebenwirkungen unter Lorlatinib. Doch konnte gezeigt werden, dass diesen mit einer Dosisanpassung befriedigend begegnet werden kann [9]. Trotz des Vorliegens der beschriebenen Nebenwirkungen verbesserte die Lorlatinibtherapie in Summe die Lebensqualität im Vergleich zur Ausgangslage.

Trotz der breiteren Wirksamkeit von Lorlatinib konnten auch unter der Therapie bereits Resistenzmechanismen identifiziert werden. Dabei entwickeln ca. 35 % der Patienten Mischresistenzmutationen im ALK-Gen unter sequenzieller Therapie mit Tyrosinkinaseinhibitoren. Analysen zum Auftreten von Resistenzen unter der Therapie von Tyrosinkinaseinhibitoren lassen vermuten, dass die Anwendung aufeinander folgender Tyrosinkinaseinhibitoren zu einer stärkeren Bildung von Mischmutationen führt mit erhöhter Therapieresistenz. Unter Umständen könnte daher eine direkte Behandlung mit Lorlatinib zu einem länger anhaltenden klinischen Ansprechen führen, da sequenzielle Mutationen weniger häufig bei therapienaiven Patienten auftreten. Die breite Wirksamkeit von Tyrosinkinaseinhibitoren der dritten Generation in der Erstlinie könnte damit die schrittweise verlaufende Anhäufung von Resistenzmutationen verhindern [10]. Es gilt also die Überlegenheit von Lorlatinib in der Erstlinie durch eine möglicherweise geringere Resistenzentwicklung und damit längeren Wirkung im Vergleich zu den Tyrosinkinaseinhibitoren der zweiten Generation nachzuweisen.

## Fazit

Lorlatinib stellt mit dem breiten Wirkungsspektrum sowie der guten intrakraniellen Wirksamkeit ein vielversprechendes Medikament in der zukünftigen Behandlung des fortgeschrittenen ALK-positiven NSCLC dar. Die hohen intrakraniellen Ansprechraten reduzieren die Notwendigkeit einer Ganzhirnbestrahlung. Jedoch werden weitere Daten benötigt, um die Überlegenheit von Lorlatinib gegenüber Tyrosinkinaseinhibitoren der zweiten Generation zu belegen und die Sicherheit des Medikaments weitergehend zu überprüfen. Angesichts der schnellen Entwicklung von Resistenzmutationen gewinnt die genetische Charakterisierung der Tumoren als Grundlage individueller Therapiekonzepte an Bedeutung.

*Johanna Richter, Kiel*
